# Type and extent of trans-disciplinary co-operation to improve food security, health and household environment in low and middle income countries: systematic review

**DOI:** 10.1186/s12889-016-3731-4

**Published:** 2016-10-18

**Authors:** Santosh Gaihre, Janet Kyle, Sean Semple, Jo Smith, Madhu Subedi, Debbi Marais

**Affiliations:** 1 Division of Applied Health Sciences, University of Aberdeen, Room 1.019 Polwarth Building, Foresterhill, Aberdeen, AB25 2ZD UK; 2 School of Biological Sciences, University of Aberdeen, Aberdeen, UK; 3 Save the Children, Kathmandu, Nepal; 4 WMS- Health Education, The University of Warwick, Coventry, UK

**Keywords:** Agriculture, Food security, Nutrition, Household air pollution, Water quality, Intervention, Health

## Abstract

**Background:**

Although linkages have been found between agricultural interventions and nutritional health, and the development of clean fuels and improved solid fuel stoves in reducing household air pollution and adverse health effects, the extent of the potential of combined household interventions to improve health, nutrition and the environment has not been investigated. A systematic review was conducted to identify the extent and type of community-based agricultural and household interventions aimed at improving food security, health and the household environment in low and middle income countries.

**Methods:**

A systematic search of Ovid MEDLINE, PUBMED, EMBASE and SCOPUS databases was performed. Key search words were generated reflecting the “participants, interventions, comparators, outcomes and study design” approach and a comprehensive search strategy was developed following “Preferred Reporting Items for Systematic Reviews and Meta-Analyses” recommendations. Any community-based agricultural and/or household interventions were eligible for inclusion if the focus was to improve at least one of the outcome measures of interest. All relevant study designs employing any of these interventions (alone/in combination) were included if conducted in Low and middle income countries. Review articles, and clinical and occupational studies were excluded.

**Results:**

A total of 123 studies were included and grouped into four intervention domains; agricultural (*n* = 27), air quality (*n* = 34), water quality (*n* = 32), and nutritional (*n* = 30). Most studies were conducted in Asia (39.2 %) or Africa (34.6 %) with the remaining 26.1 % in Latin America. Very few studies (*n* = 11) combined interventions across more than one domain. The majority of agricultural and nutritional studies were conducted in Africa and Asia, whereas the majority of interventions to improve household air quality were conducted in Latin America.

**Conclusions:**

It is clear that very little trans-disciplinary research has been done with the majority of studies still being discipline specific. It also appears that certain low and middle income countries seem to focus on domain-specific interventions. The review emphasizes the need to develop holistic, cross-domain intervention packages. Further investigation of the data is being conducted to determine the effectiveness of these interventions and whether interdisciplinary interventions provide greater benefit than those that address single health or community problems.

**Electronic supplementary material:**

The online version of this article (doi:10.1186/s12889-016-3731-4) contains supplementary material, which is available to authorized users.

## Background

Although there has been a significant improvement in global food security, still 805 million people (one in eight people) in low and middle income countries (LMIC) remain chronically undernourished [1]. According to the key findings of the Global Food Security Index 2015 [2], the rate of under nutrition is considerably higher in low and lower middle income countries (25.4 % and 16.5 % respectively) compared to high income countries (4.9 %). It is also estimated that 29.1 % and 15.5 % of children under the age of five years in lower middle income countries are either stunted or underweight. The prevalence rate is even higher in low income countries where 39.1 % of children under the age of five years are stunted and 22.6 % are underweight [2].

In addition to the health effects of food insecurity leading to poor nutrition, household air pollution from combustion of solid cooking fuels such as firewood, charcoal, etc. is the fourth leading cause of mortality in LMIC [3]. Evidence from epidemiological studies have shown that exposure to household air pollution is associated with acute respiratory tract infection, chronic obstructive pulmonary disease (COPD), cataract and lung cancer [4–6]. Likewise diarrhoea and other common infectious diseases due to poor hygiene and sanitation are also causing significant public health problems in LMIC [3].

It is evident that health is a complex phenomenon determined by multiple risk factors. Complex environmental interactions make it difficult to determine pathways to health in many communities. Food and diet is clearly an important route for exposure to pathogens, but it should not be considered in isolation, since other environmental exposures, such as household air pollution due to burning of biomass for cooking, pesticide exposure from agricultural use and polluted water for drinking, can be equally or more important to health. Food insecurity leading to poor nutrient intake is the main cause of malnutrition, but it is also dependent on other immediate causes, such as the individual’s health status [7]. Previous studies have recognised strong linkages between agricultural interventions and nutritional health [8–10] and the development of clean fuels and improved solid fuel stoves in reducing household air pollution and adverse health effects [11]. However, the scale and effectiveness of combined household interventions to improve health, nutrition and the environment has not been investigated. It is unknown whether interventions are inter-disciplinary, crossing domains of health, nutrition, agriculture and/or environment and where these interventions are being conducted. This review determined the extent and types of community-based complex agricultural and household interventions to improve food security, health status and the household environment in LMIC.

## Methods

### Search strategy

A comprehensive search strategy was developed following the recommendations in the PRISMA (Preferred Reporting Items for Systematic Reviews and Meta-Analyses) statement [12]. Key search words were generated reflecting the PICOS (participants, interventions, comparators, outcomes and study design) approach [12]. A database search of Ovid EMBASE was performed using Medical subject heading (MeSH) terms, keywords and truncations covering the potential interventions, outcomes of interest and study design (Additional file 1). The search strategy was developed by combining those search terms using appropriate Boolean operators such as AND/OR/NOT. The search strategy for Ovid MEDLINE, PUBMED and SCOPUS databases were then derived from those search terms and conducted in January 2015. In addition, web and hand searches of bibliographies of identified studies were also performed manually to identify any additional potentially eligible articles.

### Study selection and inclusion criteria

Community-based agricultural and household interventions such as the introduction of biogas, improved cook stoves, home gardening, animal husbandry, livestock farming and nutrition education were eligible to be included in this study if the focus of the intervention was to improve at least one of the outcome measures of interest (Table 1). Human studies employing any of these interventions, alone or in combination, and published after 1990, were included.

**Table 1:** Definitions of outcomes of interest measured

Outcome categories	Outcomes of interest measured
Food production	Year round of food production, production of vitamin A- rich fruits and vegetables, poultry stock and egg production, fish production, access to goat milk and other home grown foods
Food consumption	Household food security level/score, Dietary Diversity Score (DDS), consumption of food/food groups per day
Nutrient intake	Micro- and macro-nutrient intake levels
Anthropometry	Prevalence of Stunting [Weight for age Z-score (WAZ)], Wasting [height for age Z-score (HAZ)], underweight, child growth, height and weight gain
Nutrient deficiencies	Vitamin A deficiency level, Incidence/prevalence of anaemia, serum retinol concentration, serum ferritin level, haemoglobin, night blindness
Air quality	Kitchen/household/personal exposure to carbon monoxide (CO) and/or concentration of fine particulate matter of diameter < 2.5 μm (PM2.5), kitchen smoke, suspended particulate matter (PM) concentration, nitrogen dioxide concentration, ratio of food to fuel
Health	Incidence and/or prevalence of: Diarrhoeal disease; morbidity; respiratory disease symptoms (cough, runny nose, breathlessness, incidence of chronic obstructive pulmonary diseases (COPD), pneumonia); eye irritation/infection, headache. Changes in: lung function performance; cognitive performance and attention levels; quality of life
Microbial Contamination	*Thermo tolerant coliforms* (TCC) count, level of *E.coli* contamination
Hygiene and sanitation	Kitchen and hand hygiene, behaviour and knowledge of water storage, self-reported compliance
Education	Perception and knowledge of health and nutrition

The review was open to include any interventional or observational study, such as randomised control trial (RCT), cluster-randomised trial (CRT), cross-sectional study (CSS) and longitudinal studies conducted in LMIC as defined by the World Bank list of economics for 2015. As the main focus of this study was to identify community-based household interventions, clinical and occupational studies were excluded from the review. Similarly, review articles and studies from high income countries were excluded from the review.

**Fig. 1:**
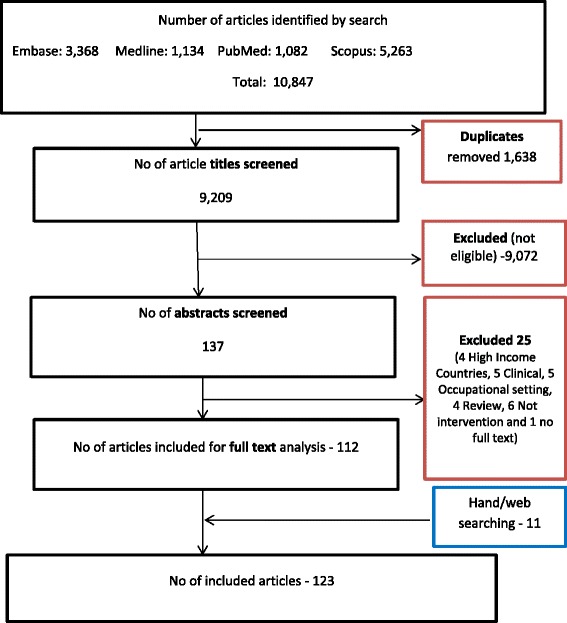
PRISMA flow diagram

All articles identified by electronic searching from the four databases were exported to a web-based bibliography and database manager namely, Refworks. The titles were merged in one database and duplicates removed (Fig. 1). The primary reviewer (SG) screened titles and selected potentially relevant abstracts following predefined inclusion/exclusion criteria. Then four further reviewers (DM, SS, JK and JS) independently examined 10 % of randomly selected titles and abstracts to ensure the accuracy of title and abstract screening process. Disagreements between reviewers were resolved through discussion and checking the full text articles. All articles deemed potentially eligible were retrieved in full text. Reference lists of included studies were also checked to identify other relevant studies.

### Data extraction and management

A standard data extraction form (Additional file 2) was designed considering the Cochrane systematic review data collection checklist [13]. The data collection form was piloted and amended prior to starting the formal data extraction.

Data from all included studies were extracted independently by three reviewers. The extracted data from 10 % of randomly selected articles was then checked independently by a second reviewer to ensure all the correct information was recorded.

### Data analysis

A narrative analysis was conducted based on interventional categorisation. Interventions were categorised according to four domains defined as follows:

- Agricultural interventions: Interventions such as home gardening and animal husbandry that have the explicit goal of improving food productivity, nutritional status, health, dietary diversity and/or food security.
- Air quality interventions: Interventions such as improved cook stove and biogas that have the clear aim of improving household air quality and occupant’s health.
- Water quality interventions: Interventions such as water filters (sand and bio sand), solar disinfection technique, water treatment using chlorine tablets alone and/or combination with sanitation health and hygiene education that have the clear aim of improving drinking water quality and health.
- Nutritional interventions: Interventions such as nutrition education, complementary food and nutritional supplements that have the clear aim of improving participants’ nutritional status, dietary diversity, and health and food security.

The studies from each interventional category were summarised in tables and narrative text provided to summarise the following aspects:

- country where the study was conducted
- sample size
- setting
- study designs followed
- types of interventions provided
- intervention duration
- outcomes of interest measured

### Assessment of methodological quality

An assessment of the validity of included studies was conducted alongside the data extraction using the Effective Public Health Practice Project (EPHPP) quality assessment tool for quantitative studies [14]. Studies were categorised as strong, moderate or weak based on their quality with regards to component ratings of selection bias, study design, confounders, blinding, data collection method, withdrawals and drop-outs and analysis.

## Results

### Identified studies

The search retrieved 10,847 unique articles (Fig. 1). After removal of 1,638 duplicates the remaining 9,209 articles were screened on the basis of title review. The first stage selection excluded 9,072 articles on the basis of predefined exclusion criteria. Studies were mainly excluded as they were conducted in high income countries, clinical or occupational settings, were not interventional studies or review articles, etc. From these 137 articles were potentially eligible for abstract screening. Finally, 112 articles met the eligibility criteria for the detailed analysis. Of the 25 articles excluded at the abstract screening stage four of them were from high income countries, five were in a clinical setting (Cl), five involved occupational settings, four were review articles, six papers were not interventional studies, and the full text of one paper was not available. Eleven additional articles were identified by hand/web searching. Finally, a total of 123 studies were included for the final review.

### Study characteristics

**Fig. 2:**
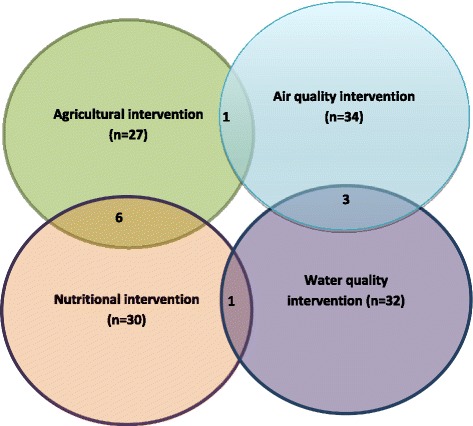
Overlapping intervention domains

Of the 123 included studies in the review, 27 (21.9 %) were agricultural interventions, 34 (27.6 %) were air quality interventions, 32 (26 %) were water quality interventions and 30 (24.3 %) were nutritional interventions (Fig. 2).

### Characteristics of agricultural interventions (*n* = 27)

Of the 27 studies (Table 2) reporting agricultural interventions, 14 projects promoted and supported home gardening and household food production or the improvement of the existing garden with micronutrient-rich fruit and vegetables. Six projects promoted animal husbandry, such as pig and poultry breeding, goat farming, fisheries and dairy production. Five studies observed the effectiveness of combined home gardening and nutrition education intervention. One promoted home gardening with animal husbandry and another, a combination of home gardening, animal husbandry and nutrition education.

**Table 2:** Characteristics of agricultural intervention studies

Study (Author and publication year)	Country	Participants (sample size, age, setting)	Study design	Intervention details (I = Intervention and C = Control)	Duration of intervention (months)	Outcome measured
Ayele Z and Peacock C; 2003	Ethiopia	210 households	CSS (Pre and post)	I: Animal husbandry: goat farming	NR	Food consumption, nutrient deficiencies
Belachew T et al. 2013	Ethiopia	2100 adolescents, 13–17 years, household	5 year Longitudinal study	I: Food production	NR	Food consumption
Bezner KR, et al. 2010	Malawi	3838 children <3 years, household	Prospective quasi- experimental study	I: Intercropping legumes and nutrition education C: Usual practice	72	Anthropometry
Bloem MW et al. 1996	Bangladesh	7341 participants, all aged, household	Intervention study	I: Home gardening	NR	Food production
Bushamuka VN, et al. 2005	Bangladesh	2,160 households	Intervention study	I: Home gardening C: Usual practice	NR	Food production, food consumption
Cabalda AB, et al. 2011	Philippines	200 households, participants aged 2–5 years	CSS (2 group comparison)	I: Home gardening (*n* = 105) C: Without home garden (*n* = 95)	NR	Food consumption
Faber M, et al. 2002,	South Africa	208 participants, aged 2–5 years, community	CSS (Pre and post)	I: Home gardening and nutrition education (*n* = 108) C: Usual practice (*n* = 100)	20	Food consumption, nutrient intake, nutrient deficiencies
Gibson RS et al. 2003	Malawi	281 households, aged 30–90 months	Intervention study	I: Multiple: Animal husbandry and home gardening (*n* = 200) C: Usual practice (*n* = 81)	12	Food consumption, anthropometry, education, nutrient deficiencies, health
Haseen F, 2007	Bangladesh	370 households, all age participants	CSS (Pre and post)	I: Home based food production, increased purchasing capacity to improve food intake and nutritional status (*n* = 180) C: Usual practice (*n* = 193)	24	Food consumption, nutrient intake
Hoorweg J, et al. 2000	Kenya	144 households, participants aged between 6–59 months	Intervention study	I: Dairy farming (*n* = 30) and dairy customers (*n* = 24) C: Usual practice (*n* = 90)	NR	Food consumption, anthropometry, income
Hop LT; 2003	Vietnam	NR	Longitudinal survey (LS) (pre and post)	I: Programs to improve pig and poultry breeding	NR	Food consumption, nutrient deficiencies
Hotz C, et al. 2012	Uganda	>10,000 households, community	Randomised control trial (RCT)	I1: *B*-carotene–rich orange sweet potato (OSP) vines with training (*n* = 293 children, 212 women) I2: Education on female and child health and promotion of OSP (*n* = 179 children, 130 women) C: Usual practice (*n* = 280 children, 213 women)	12 and 24	Nutrient intake, nutrient deficiencies
Jones KM, et al. 2005	Nepal	819 households, community	Intervention study	I: Home gardening and nutrition education (*n* = 430) C: Usual practice (*n* = 389)	36	Food consumption, education
Kalavathi S, et al. 2010	India	150 household	Intervention study (pre and post)	I: Package intervention of nutrition gardening, livestock rearing and nutrition education	36	Food production, food consumption and nutrient intake
Kerr RB, et al. 2010	Malawi	3838 participants, aged < 3 years, households	Intervention study	I: Home gardening and nutrition education (*n* = 1724) C: Usual practice	72	Anthropometry
Kidala D, et al. 2000	Tanzania	2250 household	Quasi-experimental (2 groups comparison)	I: Horticultural and nutrition education (*n* = 125 households) C: Usual practice (*n* = 125 households)	60	Nutritional knowledge, nutrient intake, nutrient deficiencies
Low JW, et al. 2007	Mozambiqu	741 children aged 13 months, household	Quasi-experimental (2 groups comparison)	I: Production of Orange-fleshed sweet potato (OFSP) and nutritional knowledge (*n* = 498) C: Usual practice (*n* = 243)	24	Nutrient intake, nutrient deficiencies
Miura S, et al. 2003	Philippines	152 women, household	CSS (pre and post)	I: Home gardening	NR	Food consumption
Murshed-e-Jahan K, et al. 2010	Bangladesh	NR	Intervention study	I: Training support to farmers on aquaculture C: Usual practice	NR	Food production, food consumption
Nielsen H, et al. 2003	Bangladesh	70 households, women of reproductive age and 5–12 years old girls	Intervention study	I: Poultry production (*n* = 35) C: Usual practice (n = 35)	12	Food production, food consumption
Olney DK, et al. 2009	Cambodia	500 households	CSS (Pre and post)	I: Home gardening (*n* = 300) C: Usual practice (*n* = 200)	NR	Food consumption, anthropometry, health
Schipani S, et al. 2002	Thailand	60 children, household	Intervention study	I: Mixed home gardening (*n* = 30) C: Non gardening (*n* = 30)	NR	Food consumption, anthropometry
Schmid M et al. 2007	India	220 participants, Child:6 to 39 months and mother > 15 years, community	CSS (pre and post)	I: Home gardening (*n* = 124) C: Without home garden (96)	96	Nutrient intake
Sha KK et al. 200,	Bangladesh	1343 participants aged <24 months, households	Longitudinal study	I: Household production and availability of rice and other fresh foods e.g. Vegetables, fish, meat	NR	Food consumption, anthropometry
Smitasiri et al. 1999	Thailand	15 communities, all age	CSS (pre and post)	I: Home gardening (seed grant) and nutrition and health messages (271) C: without home gardening (247)		Food consumption, nutrient intake
Wyatt AJ, et al. 2013	Kenya	92 households	CSS (3 group comparison)	Dairy intensification I1: Milk production >6 l per day (*n* = 31) I2: Milk production <6 l per day (*n* = 31) C: No milk production (*n* = 30)	2	Food consumption
Yakubu A, et al. 2014	Nigeria	58 households, community	CSS (pre and post)	I: Cockerel exchange programme	NR	Food production

*RCT* randomised control trial, *CSS* cross sectional study, *NR* not reported

Most of the studies were either cross sectional (*n* = 10) or intervention studies (*n* = 10) with one RCT [15]. There was a wide variation of sample sizes, ranging from 58 households [16] to >10,000 participants [15]. Similarly, duration of the studies varied; from a dairy intensifying intervention in Kenya for two months [17] to a home gardening study in India for 96 months [18]. Fourteen of these studies were conducted in Asia and the other 13 in Africa. The first home gardening study was conducted in Bangladesh in 1996 [19]. Most of these studies (*n* = 22) were conducted in a household setting and only a few in community settings.

Nineteen of these studies examined the effect of intervention on dietary diversity and improvement in food consumption, seven on food production, seven on nutrient intake, seven on nutritional deficiencies, seven on anthropometry, three on education, two on health and two on food security.

### Characteristics of air quality interventions (*n* = 34)

Of the 34 air quality studies (Table 3), four projects introduced biogas [13–20] as an alternative means of cooking fuel, 17 projects promoted improved cook stoves and 11 studies examined the effectiveness of improved stoves with chimney to improve the household air quality. One project evaluated the impact of improved cook stoves with solar water disinfection and hand hygiene [21], and another looked at an improved cook stove intervention with biogas fuel and solar heaters [20].

**Table 3:** Characteristics of air quality intervention studies

Study (Author and publication year)	Country	Participants (sample size, age, setting)	Study design	Intervention details (I = Intervention and C = Control)	Duration of intervention (months)	Outcome measured
Alexander D, et al. 2013	Bolivia	31 household	Intervention study (pre and post)	I: Improved cook stoves with chimney (*Yanalo Cookstoves*)	12	Air quality, health
Burwen J and Levine DI; 2012	Ghana	768 household	RCT	I: Improved cook stoves with chimney (*n* = 402) C: Traditional biomass stoves (usual practice) (*n* = 366)	2	Air quality, health, stove usages
Chengappa C, et al. 2007	India	60, household	Paired, before and after study	I: improved cook stoves (*Sukhad*)	12	Air quality
Clark LM, et al. 2009	Honduras	79 participants, mean age 43.2 years, household,	CSS (pre and post)	I: Improved cook stoves with chimney (*n* = 38) C: Traditional cook stoves (*n* = 41)	3	Air quality, health
Chowdhury Z et al. 2012	China	30 household	CSS (pre and post)	I: Improved stoves along with biogas burners and solar heaters	2	Air quality
Commodore AA, et al. 2013	Peru	84 participants household	Community-RCT (C-RCT)	I: Improved cook stoves (*OPTIMA*) (*n* = 39) C: Traditional biomass stove, NGO Stoves, self-improved stove (*n* = 45)	3	Air quality, health
Cynthia AA, et al. 2008	Mexico	34 households,	Randomised trial	I: Improved cook stoves (*n* = 60)	1	Air quality
Diaz E, et al. 2008	Guatemala	180 women, mean age 27.8 years, household	RCT	I: Improved cook stoves with chimney (*Plancha*) (*n* = 89) C: Traditional biomass stove (usual practice) (*n* = 91)	26	Air quality, health
Diaz E, et al. 2007	Guatemala	504 women, 27.7 years, household	RCT	I: Improved cook stoves with chimney (*Plancha*) (*n* = 259) C: Traditional biomass stove (usual practice) (*n* = 245)	18	Air quality, health
Dohoo C, et al. 2012	Kenya	62 women, household	CSS (comparison between 2 groups)	I: Biogas (*n* = 31) C: Traditional biomass stove (*n* = 31	2	Health
Ezzati M, et al. 2000	Kenya	38 households	Intervention study	I: Improved cook stoves	1	Air quality
Fitzgerald C, et al. 2012	Peru	57 participants, mean age 33 years, household	Intervention study (pre and post)	I: Improved cook stoves (*n* = 26 for PM2.5 and 25 for CO)	5	Air quality
Garfi M, et al. 2012	Peru	12 households	Intervention study	I: Low-cost tabular biogas digester	NR	Food production, air quality
Harris SA, et al. 2010	Guatemala	4000, household	Intervention study (pre and post)	I: Improved cook stoves C: Traditional biomass stove (usual practice)	48	Health
Hartinger SM, et al. 2012	Peru	115 households, household,	Intervention study (pre and post)	I: Multiple intervention; improved cook stoves, solar water disinfection and hand hygiene	5	Air quality, hygiene and sanitation, health
Jary HR, et al. 2014	Malawi	51 Women, mean age 38.1 years, households	RCT	I: Improved cook stoves (*n* = 25) C: Traditional biomass stove (usual practice) (*n* = 26)	2	Air quality, health
Katwal H, Bohara AK; 2009	Nepal	461 households	Intervention study	I: Biogas digester	NR	Air quality, health, Food production
Khushk WA, et al. 2005	Pakistan	159 women, mean age 43.27 (I) and 36.18 (C) years, household	CSS (comparison between 2 groups)	I: Improved cook stoves (*n* = 45) C: Traditional biomass stove (usual practice) (*n* = 114)	2	Air quality, health
Li Z, et al. 2011	Peru	57 households, participants aged 18–45 years, household	Intervention study (pre and post)	I : Improved cooking stove with chimney	3 weeks	Air quality
McCracken JP, et al. 1998	Guatemala	11, household	CSS (comparison between 2 groups)	I: Improved cook stoves (*n* = 6) C: Traditional biomass stove (usual practice) (*n* = 5)	NR	Air quality
McCracken JP, et al. 2011	Guatemala	534 Households	RCT	I: Improved stove with Chimney (*n* = 49) C: Traditional open fire stoves (*n* = 70)	16	Air quality, health
Mukhopadhyay R, et al. 2012	India	32 women, mean age 32 years, household	CSS (pre and post)	I: Improved cook stoves C: Traditional open fire biomass stove (usual practice)	3	Air quality, acceptability and usage
Ochieng CA, et al. 2012	Kenya	104 Women, household	CSS (comparison between 2 groups)	I: Improved stoves without chimney (*n* = 49) C: Traditional stoves (*n* = 45)	6	Air quality
Oluwole O, et al. 2013	Nigeria	59 participants, mothers 43 years and children 13 years, household	CSS (pre and post)	I: Improved stoves	12	Air quality, health
Pandey MR, et al. 1990	Nepal	20 households	Intervention study	I: Improved cook stoves (*n* = 20)	5	Air quality
Riojas-Rodriguez, et al. 2011	Mexico	47 women, mean age 28 years, household	RCT	I: Improved cook stoves fitted with chimney (*Patsari stoves*) (*n* = 30) C: Traditional stoves (*n* = 17)	12	Air quality
Romieu I, et al. 2009	Mexico	528 women, mean age 26.3 (I) and 25.5 (C) years, household	RCT	I: Improved cook stoves fitted with chimney (*Patsari stoves*) (*n* = 273) C: Traditional stoves (*n* = 255)	10	Health
Schilmann A, et al. 2014	Mexico	559 children <4 years, household	RCT	I: Improved cook stoves fitted with chimney (*Patsari stoves*) (*n* = 287) C: Traditional stoves (*n* = 272)	10	Health
Singh A, et al. 2012	Nepal	47 households, all aged participants	CSS (pre and post)	I: Improved mud stoves	12	Air quality, health
Singh S, et al. 2014	India	75 household	CSS (comparison between 2 groups)	I: Improved stoves C: Traditional stoves	2	Air quality
Smith KR, et al. 2011	Guatemala	534 households, participants aged <4 months at baseline	RCT	I: Improved wood stove with chimney (*n* = 265) C: Open wood fires (*n* = 253)	14	Health
Wafula EM, et al. 2000	Kenya	400 households, women aged 15–60 years and children <5 years	Intervention study (pre and post)	I: Improved cook stoves (*n* = 200) C: Traditional three-stone stoves (*n* = 200)	120	Health
Zhou Y, et al. 2014	China	996 participants, aged > 40 years, household	CSS (comparison between 2 groups)	I: Biogas digester and improved kitchen ventilation (*n* = 740) C: Traditional biomass stove (usual practice) (*n* = NR)	108	Air quality, health
Zuk M, et al. 2007	Mexico	53 household	CSS (pre and post)	I: Improved cook stoves (*Patsari stoves*)	5	Air quality

*RCT* randomised control trial, *CSS* cross sectional study, *NR* not reported

Most of the studies provided data either on pre and post or between group comparisons with nine randomised control trial. The sample sizes of the studies ranged from 11 [22] to 4,000 households [23]. The duration of the study also varied considerably; a Peru cook stove project lasted for 3 weeks [24], while one vented stove project in the highlands of Guatemala collected data for 48 months [23]. The majority of the studies (*n* = 18) were conducted in South America, nine were in Asia, with the other seven in African countries. The first cook-stove intervention study was conducted in Nepal in 1990 [25]. All of these studies were conducted in household settings.

Almost all of the studies (28 out of 34) examined the improvement in household air quality parameters such as particulate matter and carbon monoxide concentrations. Twenty studies assessed the impact of the intervention on participants’ health outcomes such as incidence of pneumonia, acute respiratory infections (ARI), conjunctivitis and lung function, and three examined the impact on food production.

### Characteristics of water quality interventions (*n* = 32)

Of the 32 water quality intervention studies (Table 4), 12 were water filter interventions; nine were chlorine tablets/solutions interventions, seven were Solar disinfection; two were hand water pumps along with hygiene education and latrine construction interventions [26]; one was a health, hand hygiene, water quality and sanitation educational intervention [27]; one involved disinfection tablets along with sanitation and hygiene education [28]; one was a water disinfection stove [29] and one a filter along with improved cook stove [30].

**Table 4:** Characteristics of water quality intervention studies

Study (Author and publication year)	Country	Participants (sample size, age, setting)	Study design	Intervention details (I = Intervention and C = Control)	Duration of intervention (months)	Outcome measured
Boisson S, et al. 2010	Democratic Republic of Congo (DRC)	240 household (1,144 participants mean age 39.1 years)	RCT	I: Lifestraw family filter (*n* = 120 households, 546 participants) C: Placebo filter (*n* = 120 households, 598participants)	15	Microbial contamination, health
Boisson S, et al. 2009	Ethiopia	313 households, 6 months and over, household	RCT	I: Life straw personal filter to be used for ingesting of untreated water both at home and away from home (*n* = 155) C: Usual practice (*n* = 158)	5	Microbial contamination, health
Boisson S, et al. 2013	India	2,163 household (2,986 children <5 years)	RCT	I: NaDC tablets^b^ (*n* = 1080) C: Placebo (*n* = 1083)	12	Microbial contamination, health
Brown J et al. 2008	Cambodia	180 households, all age participants	RCT	I: One of following: Ceramic water purifier (CWP) (*n* = 60) and Iron-rich ceramic water purifier (CWP-fe) (*n* = 60) C: Usual practice (*n* = 60)	5.5	Microbial contamination, health
Clasen T.F et al. 2006	Bolivia	60 households (317 individuals), all age, household	RCT	I: Water purification filter (20 households; 210 individuals) C: Usual practice (40 households; 107 individuals)	5	Microbial contamination, health
Clasen T, et al. 2007	Bangladesh	100 households, 555 participants of any age group	RCT	I: 67-mg NADCC tablets^b^ designed to treat 20–25 L of water (n= 50 households; 279 participants) C: Placebo consisting of tablets of the same colour, size and packaging (*n* = 50 households, 276 participants)	4	Microbial contamination
Clasen T, et al. 2005	Columbia	140 household	RCT	I: Ceramic Water filter (*n* = 76 households, 415 participants) C: Usual practice (*n* = 64 households, 265 participants)	6	Microbial contamination, health
Christen A, et al. 2009	Bolivia	2 household (27 proxy household for air quality)	CSS (pre and post)	I: Water disinfection stove (WADIS)	6	Water quality, Microbial contamination, air quality, health
Conroy R, et al. 1996	Kenya	206 children age 5–16 years, household	RCT	I: SODIS bottle (*n* = 108) C: Only water bottle and suggested to use indoor (*n* = 98)	3	Health
Crump JA, et al. 2005	Kenya	605 households (6650 participants)	Cluster- RCT	I1: Flocculant- disinfectant intervention (*n* = 201 households,2124 participants) I2: Sodium hypochlorite intervention (*n* = 203 households, 2249 participants) C: Usual practice (*n* = 201 households, 2277 participants)	4 (20 weeks)	Microbial contamination, health
Davis J, et al. 2011	Tanzania	248 households, participants aged <5 years	Experimental field study	I: One of following 4 intervention: 1) Information on strategies to reduce water and sanitation related illness (*n* = 79) 2) Information as per 1 plus water quality tests (*n* = 84) 3) Information as per 1 plus hand-rinse test results (*n* = 90) 4) information as per 1 plus water and hand rinse results (*n* = 81)	4	Microbial contamination, hygiene and sanitation
Du Preez M, et al. 2008	Zimbabwe and South Africa	115 households, participants aged between 12 to 24 months	RCT	I: Ceramic water filter (*n* = 60) C: In-house water filter (*n* = 58)	6	Health
Du Preez M, et al. 2010	South Africa	649 households, 6 months to 5 years, household	RCT	I: SODIS^a^ bottles to be used to provide drinking water at all times and as much as possible drink directly from the bottle (*n* = 297) C: Usual practice (*n* = 267)	12	Microbial contamination, health
Fabiszewski de Aceituno AM, et al. 2012	Honduras	195 participants aged <5 years, household	RCT	I: Plastic Bio sand filters, a narrow mouth gallon (20 L), water jug and general education on hygiene and sanitation (*n* = 90 households, 532 participants) C : Usual practice (*n* = 86 households, 488 participants)	10	Microbial contamination, health
Graf J, et al. 2010	Cameroon	2,193 households, participants aged <5 years	CSS (pre and post)	I: SODIS bottles for water purification	10	Health
Garrett V, et al. 2008	Kenya	555 households (960 children aged <5 years)	RCT	I: Sodium hypochlorite water disinfection solution and storage containers and hygiene and sanitation education (*n* = 366) C: Usual practice (*n* = 189)	2 (8 weeks)	Microbial contamination, health
Habib MA, et al. 2013	Pakistan	18,244, participants, household	Cluster-RCT	I: Diarrhoea pack (two packets of low osmolality ORS, one strip of Zinc tablets, two packets of water purification sachet and a leaflet with educational materials) (*n* = 9,581) C: Usual practice (*n* = 8,663)	12	Health
Henry FJ et al. 1990	Bangladesh	44 children, 6–23 months, community	Intervention Study	I: Latrine construction and hygiene education (*n* = 41) C: Usual practice (*n* = 43)	6	Health
Henry FJ et al. 1990	Bangladesh	92 participants, 6–18 months, household	Intervention study	I: Hand pumps, latrine construction and hygiene education (44) C: Hand pumps only (48)	6	Health
Lindquist ED, et. al; 2014	Bolivia	1,198 participants, household	Cluster-RCT	I1: A household level hollow fiber filter (*n* = 330) I2: Education (behaviour change communication) (*n* = 302) I3: Filter and education (*n* = 285) C: Life skills and attitudes and family responsibility message (*n* = 279)	3	Health
Luby,AP, et al. 2006	Pakistan	1340 households, all age participants	RCT	I: One of following intervention: 1) diluted bleach and a water vessel provided (*n* = 265) 2) soap and hand washing promotion provided (*n* = 262) 3) flocculent disinfectant water treatment and water vessel provided (*n* = 262) 4) flocculent-disinfection, soap and hand washing promotion provided (*n* = 266) C: Usual practice (n = 282)	9	Health
Mausezahi D et al. 2009	Bolivia	484 households, participants aged <5 years	RCT	I: SODIS bottles (*n* = 255 households; 376 children) C: Usual practice (*n* = 200 households; 349 children)	14	Health
Opryszko MC et al. 2010	Afghanistan	1514 households, all age participants, household	RCT	I: Multiple intervention; liquid chlorine with a water vessel (299 households), hygiene education (233 households), improved tube well (308 households) and combination of all (261 households) C: Usual practice (*n* = 292)	17	Diarrhoeal incidence
Quick RE et al. 1996	Bolivia	42 household	Intervention study (pre and post)	I1: 20 l narrow mouthed water vessel and the calcium hypochlorite solution (*n* = 15) I2: 20 l narrow mouthed water vessel (*n* = 15) C: Usual practice (*n* = 12)	9 weeks	Microbial contamination,
Quick RE, et al. 1998	Bolivia	127 households	RCT	I: Water disinfection solution and storage vessels (*n* = 64 households, 400 individuals) C: Usual practice (*n* = 63 households, 391 individuals)	8	Microbial contamination, health
Ram PK, et al. 2007	Madagascar	242 households, participants aged 0–90 year	Intervention study	I: Water chlorination tablet and Jerrycan for water storage	NR	Education and self-reported compliance
Rangel JM, et al. 2003	Guatemala	100 households	RCT	I1: Chlorine bleach and 20 l narrow mouthed water vessel (*n* = 20) I2: Combined product ^c^ in narrow mouthed water vessel (*n* = 20) I3: Combined product ^c^ with customised vessel (*n* = 20) I4: Combined product ^c^ in traditional vessel (*n* = 20) C: Traditional vessel (*n* = 20)	1 (4 weeks)	Microbial contamination, health
Rose A et al. 2006	India	200 children, participants aged <5 years, household	RCT	I: SODIS bottles for water purification plus diarrhoea prevention and treatment education (*n* = 100) C: Diarrhoeal prevention and treatment education only (*n* = 100)	6	Health
Rosa G, et al. 2014	Rwanda	566 households	RCT	I: Life straw family 2.0 filter and one improved stove (*Eco Zoom Dura*) (*n* = 285) C: Usual practice (*n* = 281)	5	Water quality, air quality
Stauber CE, et al. 2009	Dominican Republic	187 households, all aged participants	RCT	I: Plastic Bio Sand filters (*n* = 81 households, 447 participants) C : Usual practice (*n* = 86 households, 460 participants)	10	Microbial contamination, health
Stauber CE, et al. 2011	Cambodia	189 households, participants aged <5 years	RCT	I: Plastic Bio Sand filters (*n* = 90 households, 546 participants) C : Usual practice (*n* = 99 households, 501 participants)	6	Microbial contamination, health
Tiwari SS, et al. 2009	Kenya	59 household	RCT	I: Concrete Bio sand Filter and instruction on filter use (*n* = 30) C: Usual practice (*n* = 29)	6	Microbial contamination, health

*RCT* randomised control trial, *CSS* cross sectional study, *NR* not reported, ^a^SODIS: Solar Disinfection method, ^b^NADCC tablets: Sodium Dichloroisocyanurate tablets, ^c^ Combined product: a product incorporating precipitation, coagulation, flocculation and chlorination technology

Most of the studies were RCT (*n* = 25) or intervention studies (*n* = 4). The sample sizes of the studies ranged from 2 [29] to 2,193 households [31] and the interventions were delivered over periods of 2 [29] to 15 [32] months. Nine studies were conducted in South America, 10 in Asia and the remaining 13 in African countries. All of these studies were conducted in household settings.

Twenty-seven of these studies looked at the impact of intervention on health especially on the incidence/prevalence of diarrhoeal diseases; 20 on microbial contaminations and water quality; two studies examined the level of knowledge and self-compliance, two investigated air quality and one hygiene and sanitation.

### Characteristics of nutrition Interventions (*n* = 30)

Of the 30 nutrition intervention studies included in the review (Table 5), 11 studies were supplementary food and vitamin interventions, 13 nutrition education interventions, five nutrition education together with complementary food interventions, two combined interventions of nutrition education and home gardening [33, 34] and one combined package intervention of health care, nutrition education, water and sanitation [35].

**Table 5:** Characteristics of nutrition intervention studies

Study (Author and publication year)	Country	Participants (sample size, age, setting)	Study design	Intervention details (I = Intervention and C = Control)	Duration of intervention (months)	Outcome measured
Ali D et al. 2013	Bangladesh, Vietnam, Ethiopia	2356 (Ethiopia), 3075 (Vietnam), 3422 (Bangladesh) households, participants aged 6 monthsnths-5 years	CSS	I: Nutrition education	NR	Food consumption and anthropometry
Chow J, et al. 2010	India	participants aged 1–4 years, household	Intervention study	I: High dose vitamin A supplementation, Industrial fortification of mustard oil and GM fortification of mustard oil and seed	NR	Health
Creed-Kanashiro H et al. 2003	Peru	42 participants, aged 12–51 years, community	Interventional study (pre and post)	I: Nutrition education	NR	Nutrient deficiencies, education
Darapheak C, et al. 2013	Cambodia	6202 participants, aged 12–59 months, household	CSS (post intervention only)	I: Animal source food group C: Non animal source food group	NR	Anthropometry, health
English RM, et al. 1997	Vietnam	720 children <6 years, community	CSS (2 groups)	I: Home gardening and nutrition education (*n* = 469) C: Usual practice (*n* = 251)	24-36	Nutrient intake, health
Faber M, et al. 2002	South Africa	208 participants, aged 2–5 years, community	CSS (Pre and post)	I: Home gardening along with nutrition education (*n* = 108) C: Usual practice (*n* = 100)	20	Nutrient intake
Fenn B et al. 2012	Ethiopia	5552 participants, 6–36 monthsnths, household	CSS (pre and post)	I: Multiple intervention; health care, nutrition education, water and sanitation (4124) C: Protective safety net programme (1428)	30	Anthropometry
Gibson RS et al. 2003	Malawi	281 participants, aged between 30–40 months, household	Quasi- experimental	I: Complementary foods (*n* = 200) C: Usual practice (*n* = 81)	6	Food consumption, nutrient intake, anthropometry
Grillenberger, et al. 2006	Kenya	498 participants, mean age 7.4 years	RCT	I: Three supplementary foods groups: meat (*n* = 134), milk (*n* = 144) and energy (veg oil) supplied as a school snack in a maize stew (*n* = 148) C: Usual practice (*n* = 129)	24	Anthropometry
Grillenberger, et al. 2006	Kenya	554 participants, mean age 7.4 years	RCT	I: Three supplementary foods groups: meat (*n* = 134), milk (*n* = 144) and energy (veg oil) supplied as a school snack in a maize stew (*n* = 148) C: Usual practice (*n* = 129)	24	Nutrient intake, anthropometry
Imran M, et al. 2014	India	245 participants, aged 2–4 years, community	Intervention study	I: Nutrition education along with supplementary nutrition and supervision	12	Anthropometry
Kabahenda M, et al. 2011	Uganda	89 children <4 years, household	RCT	I: Nutrition education (*n* = 46) C: Sewing classes (*n* = 43)	12	Food consumption, nutrient deficiencies
Khan A Z et al. 2013	Pakistan	586 participants, aged 6 mo- 8 years, household	Intervention study (pre and post)	I: Nutrition education	3	Food consumption, anthropometry
Kilaru A, et al. 2005	India	242 infants aged 5–11 months, household	Intervention study	I: Nutrition education (*n* = 173) C: No nutrition education (*n* = 69)	36	Food consumption, Anthropometry
Lanerolle P and Atukorala S, 2006	Sir Lanka	229 adolescent girls aged between 15–19 years, household	Intervention study (pre and post)	I: Nutrition education	10 weeks	Nutrition knowledge, food consumption, nutrient deficiencies
Lartey A et al. 1999	Ghana	216 participants, aged 6–12 months, households	RCT	I: One of following complementary fortified foods: Weanimix (W) a combination of soybeans, maize and groundnuts, Weanimix plus minerals and vitamins (WM), Weanimix plus fish powder (WF) and Koko plus fish powder (KF) (*n* = 208) C: Usual practice (n = 465)	6	Anthropometry
Moore JB, et al. 2009	Nicaragua	182 adolescents and 67 mothers, community	Longitudinal study (pre and post)	I: Nutrition education	48 for girls and 24 for mothers	Nutritional knowledge, nutrient deficiencies
Pawloski LR and Moore JB; 2007	Nicaragua	186 adolescent girls aged 10–17 years, community	Intervention study (pre and post)	I: Nutrition education	36	Nutritional knowledge, Anthropometry, nutrient deficiencies
Phawa S, et al. 2010	India	370 mothers of children aged 12–71 months, community	Intervention study (2 groups)	I: Nutrition and health education (*n* = 195) C: Usual practice (*n* = 175)	9	Health
Pant CR, et al. 1996	Nepal	40,000 children aged 6–12 months	Intervention study (pre and post)	I: Mega dose vitamin A capsules and nutrition education C: Usual practice	24	Health, nutrient deficiencies
Rivera JA, et al. 2004	Mexico	650 children aged <12 months, household	Randomised crossover study	I: Nutrition Education along with micronutrient- fortified foods (*n* = 373) C: Cross over intervention group (*n* = 277)	24	Anthropometry, nutrient deficiencies
Roy SK, et al. 2005	Bangladesh	282 children aged 6–24 months, household	RCT	I1: Intensive nutrition education twice a week I2: Intensive nutrition education and supplementary food C: Nutrition education from community nutrition promotors	3	Food consumption Anthropometry, Nutrient intake, Education
Salehi M, et al. 2004	Iran	811 children aged <5 years, household	Intervention study (2 groups)	I: Nutrition education (*n* = 406) C: Usual practice (*n* = 405)	12	Anthropometry, Food consumption
Santos I, et al. 2001	Brazil	424 participants, aged <18 months, community	RCT	I: Nutritional counselling (*n* = 218) C: Usual practice (*n* = 206)	One off training	Anthropometry
Sazawal S, et al. 2010	India	633 participants, 1–4 years, community	RCT	I: Micronutrient fortified milk (*n* = 316) C: Non-fortified milk (*n* = 317)	12	Anthropometry and nutrient deficiencies
Sekartini R et al. 2013	Indonesia	54 participants, aged between 5–6 years, household	RCT	I: Four different complementary milks products; Std GUM, Iso-5 GUM, Iso-5 LP GUM, Iso-2 · 5 GUM	2	Health
Siekmann JF et al. 2003	Kenya	555 participants aged between 5–14 years	RCT	I: Three supplementary foods groups: meat (*n* = 134), milk (*n* = 144) and energy (veg oil) supplied as a school snack in a maize stew (*n* = 148) C: Usual practice (*n* = 129)	12	Food consumption, nutrient intake
Serkatini R et al. 2013	Indonesia	54 participants, aged 5–6 years, household	Cross over study	I: Four different growing up milk (GUM) products – Standard GUM, Std GUM with 5 g isomaltulose per serving (Iso-5 GUM0, Iso-5 GU with lowered protein content (Iso-5 LP GUM), Std GUM with 2.5 g isomaltulose in combination with other vitamins and minerals (Iso 2.5 GUM)	2	Health
Vitolo M R et al. 2008	Brazil	500 individuals, all age, household	RCT	I: Breastfeeding and weaning counselling and complementary foods (163 mothers baby pairs) C: No dietary advice given (234 mother-baby pairs)	6	Health
Walsh CM, et al. 2002	South Africa	815 children aged 2 to 5 years, household	Intervention study (2 groups)	I: Nutrition education plus food aid C: Food aid only	24	Anthropometry, nutrient deficiencies

*RCT* randomised control trial, *CSS* cross sectional study, NR: Not reported

Most of the studies (*n* = 18) were intervention studies (pre and post or two group comparison), ten RCT, one randomised crossover study and one crossover trial. The sample sizes of the studies ranged from 42 [36] to 40,000 [37] participants. The duration of the study also varied; from a once-off nutrition counselling training [38] to a 48 months nutrition education intervention in Nicaragua [39]. Just over half of the studies (*n* = 16) were conducted in Asia, nine in Africa and the other six in South American countries. Majority of these studies (*n* = 17) were conducted in a household settings with some in community settings.

Eighteen of the nutrition intervention studies assessed the impact of intervention on nutritional status such as growth, prevalence of stunting (low height-for-age), underweight (low weight-for-age), and wasting (low weight-for-height), 10 studies assessed food consumption and dietary diversity, nine studies assessed the impact on nutrient deficiencies, eight studies looked at health status, six at nutrient intake, five at health and nutritional knowledge, two at feeding practice and one assessed food security.

### Methodology quality

Of the 123 included studies, eight studies failed to provide sufficient detail to assess their methodological quality. Information of study selection, withdrawals, blinding and confounders were particularly under-reported in the majority of studies. Because of the nature of the intervention, it was assumed that no blinding was imposed in some studies and they were therefore categorised into moderate quality study. The most common methodological problems among the weak studies were in selection bias, confounders, reliability and validity of data collection tools and blinding.

## Discussion

According to our knowledge, this systematic review is the first to explore the cross-domain overlapping of multidisciplinary research projects in agriculture, nutrition, air quality and water quality. It is obvious that there is a lot of work being done in this area but from this review it clear that there is variation in not only the type of intervention, study type, sample size, duration and setting, but also in the outcome measured.

Although a wide variety of agricultural interventions such as home gardening and animal husbandry were conducted to improve household food productivity and food consumption, this review also confirms the findings of previous reviews that only few studies were measuring the impact of those interventions on nutritional status [8–10]. Of those projects that did look at the impact of agricultural intervention on nutrition, seven examined the impact on nutrient intake, nutrient deficiencies and anthropometry. In general it is predictable that increased production and consumption of food leads to better nutrition, but due to variation in study design, duration and outcome of interest measured among the included studies, it doesn’t look likely to obtain pooled estimate for studies which look at impact of intervention on nutritional health.

While looking at the air quality interventions, it is evident that interventions to improve cook stoves are the most popular interventions (83 %) and are widely being used in all over the world. This may provide the enough roofs to perform the meta-analysis. Some biogas interventions (*n* = 4) [20, 40–42] have been conducted to measure the multiple benefits of intervention on indoor air quality and food production (using bio-slurry). However, as they refer to different outcome measures and are measured in different ways, the available evidence does not look strong enough to perform the comprehensive analysis.

It was identified that water purification filter interventions were the most popular (*n* = 12) interventions for treatment of drinking water quality in LMIC. Other interventions such as chlorine tablets or solution (*n* = 9) and solar disinfection (*n* = 7) are also common in this region. Randomised controlled trial study design was the most popular among the water quality intervention as the vast majority (78 %) of the research project applied this method. So, it is more likely that effects on the drinking water quality can be summarised across studies.

Nutrition education (*n* = 13) and supplementary food and vitamin (*n* = 11) interventions were the most popular nutritional intervention in LMIC. Some intra-domain combined interventions of nutrition education and supplementary foods (*n* = 5) have also been piloted in some low and middle income countries to determine the impact of intervention on dietary diversity and nutrient intake.

The main finding of this review is that the vast majority (91 %) of the academic research on agricultural, nutrition and the environmental studies are simple and discipline specific with substantially fewer (*n* = 11) combined interventions across domains and the result is consistent with previous domain specific reviews [7, 43]. Only six studies looked at the combined impact of agricultural and nutrition education interventions, three on air and water quality interventions, one study examined the impact of a combination of agricultural and air quality interventions and one was a combined water quality and nutritional intervention. Although poor nutrition and household air pollution are the leading cause of mortality in LMIC [3], this review did not find any studies examining the impact of a combination of air quality and nutritional interventions on health. It is also striking that none of these studies investigating the combined impact of agricultural and drinking water quality interventions on human health. The evidence reviewed here shows that silo mentality is still inherent in academic research.

**Fig. 3:**
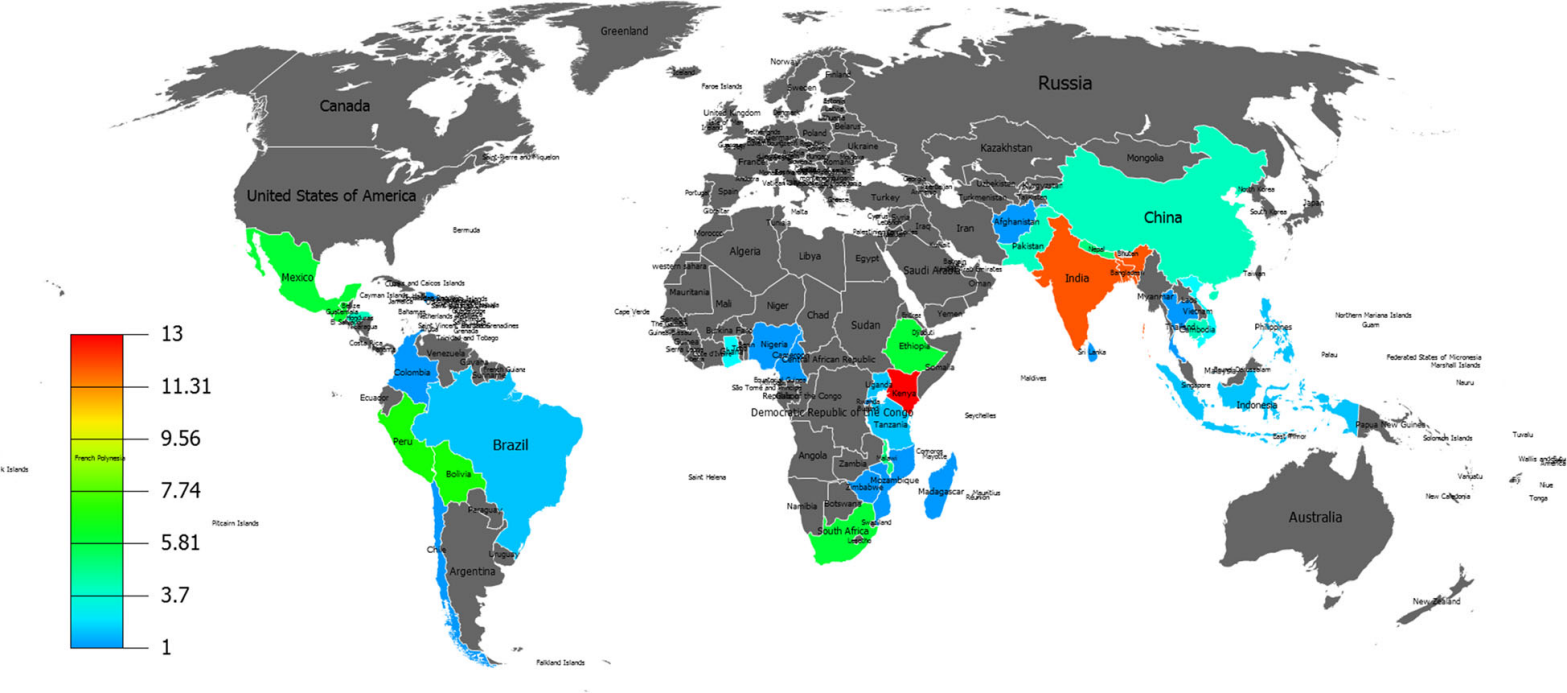
Global map highlighting the regional focus of included studies

Another interesting finding of this review is that certain LMIC regions seem to focus on domain-specific interventions, with most studies in Kenya and India and only a small number in other countries (Fig. 3). Asian and African countries were the most common regional target for agricultural and nutritional studies. More than half of the agricultural (52 %) and nutritional (53 %) interventions were conducted in Asian countries with the majority of them in south Asian countries. Similarly, 48 % of agricultural and 30 % of nutritional studies were conducted in Africa with the majority of them focussed in sub-Saharan African countries such as Kenya, Ethiopia and South Africa. The majority of water quality interventions were conducted in Africa (40.6 %) followed by Asia (31.3 %) and Latin America (28.1 %). However, the majority (53 %) of interventions to improve household air quality were conducted in Latin American countries particularly in Guatemala, Peru and Mexico. This restricts the generalisability of the findings to other LMIC.

### Strengths and limitations of the study

The main strength of this review is the application of a comprehensive search strategy through four databases to capture all potentially relevant peer reviewed articles. One hundred and twenty three articles representing the four different intervention domains provide ample evidence to understand the current research gap in interdisciplinary research. The use of independent reviewers throughout the review process further strengthened the methodological quality.

The main limitation of this study is that as only peer reviewed journal articles were included in this review, there is a chance of missing those studies published in developmental organisations’ reports and bulletins (publication bias). Additionally, this review focused on household and community-based studies, so there is a chance of missing some useful studies conducted in clinical settings.

## Conclusion

In conclusion, it is evident that very little interdisciplinary research has been conducted with the majority of studies on agriculture, nutrition and the environment being discipline specific. It also seems that certain LMIC regions seem to focus on domain-specific interventions. Although a wide variety of study designs have been implemented to measure the impact of agricultural, nutrition and air quality interventions on respective outcomes of interest measured, there is still not sufficient evidence which utilises robust randomised or quasi-experimental study design.

Therefore, this review emphasizes that future research needs to focus on multi-disciplinary complex interventions with standardised outcome measures. Also, rigorous research across disciplines and sharing expertise across regions is a necessity. The next phase of this review (Meta-analysis) will identify whether eliminating silos of discipline specific research can bring a significant improvement or not.

## Additional files

Additional file 1: Ovid Embase Search Strategy. (DOCX 16 kb)
Additional file 2: Data Extraction Sheet. (DOCX 21 kb)

## Abbreviations

ARI: Acute respiratory infections
C: Control group
CO: Carbon monoxide
COPD: Chronic obstructive pulmonary disease
CRT: Cluster-randomised trial
CSS: Cross-sectional study
DDS: Dietary diversity score
HAZ: Height for age Z-score
I: Intervention group
LMIC: Low and middle income countries
MeSH: Medical subject heading
NADCC tablets: Sodium dichloroisocyanurate tablets
NR: Not reported
PICOS: Participants, interventions, comparators, outcomes and study design
PM2.5: Fine particulate matter of diameter < 2.5 μm
PRISMA: Preferred reporting items for systematic reviews and meta-analyses
RCT: Rrandomised control trial
SODIS: Solar disinfection method
TCC: Thermo tolerant coliforms
WAZ: Weight for age Z-score
