# Supplementary material for: Type and extent of trans-disciplinary co-operation to improve food security, health and household environment in low and middle income countries: systematic review
Source: BMC Public Health. 2016 Oct 18;16:1093. doi: 10.1186/s12889-016-3731-4 (PMC5070145; doi:10.1186/s12889-016-3731-4)
Supplement: Additional file 1: — Ovid Embase Search Strategy. (DOCX 16 kb) [file 12889_2016_3731_MOESM1_ESM.docx]

**Additional file 1: Ovid Embase Search Strategy**

| **Interventions** | **Outcomes** |
| --- | --- |
| 1. agriculture/  2. agricultur$.ti,ab.  3. livestock/  4. livestock$.ti,ab.  5. animal husbandry/  6. (animal adj1 husbandry$).ti,ab.  7. (vegetable adj1 garden$).ti,ab.  8. (vegetable adj1 plot$).ti,ab.  9. (home adj1 garden$).ti,ab.  10. biogas/  11. (bio-gas or (bio adj1 gas) or biogas$).ti,ab.  12. stove$.ti,ab.  13. (cook$ adj1 stove$).ti,ab.  14. (fire adj1 stove$).ti,ab.  15. (improved adj1 stove$).ti,ab.  16. water management/  17. (water adj1 (purification or management)).ti,ab.  18. nutrition education/  19. (nutrition adj1 education).ti,ab.  20. farming.ti,ab.  21. or/1-20 | 22. (food adj1 insecurity).ti,ab. or food insecurity/  23. (nutritional adj1 status).ti,ab.  24. nutritional assessment/ or food intake/ or dietary intake/ or nutritional status/  25. (diet$ adj1 diversity).ti,ab.  26. (food adj1 consumption).ti,ab.  27. lung disease/ or asthma/ or respiratory tract disease/ or lung function/ or chronic obstructive lung disease/  28. (respiratory adj1 health).ti,ab.  29. particulate matter/ or indoor air pollution/ or air quality/ or air pollutant/  30. (indoor adj1 air adj1 (qualit$ or pollution)).ti,ab.  31. food security/  32. (food adj1 security).ti,ab.  33. (food adj1 production).ti,ab.  34. (water adj3 quality).ti,ab. or water quality/  35. (water adj1 pollution).ti,ab.  36. (soil adj1 (quality$ or pollution)).ti,ab. or soil pollution/ or soil quality/  37. (diarrhoea or diarrhea).ti,ab. or diarrhea/  38. or/22-37 |
| **Study design** | **Inclusion and exclusion criteria** |
| 39. Intervention$.ti,ab. or intervention study/  40. follow up/ or (Follow adj3 stud$).ti,ab.  41. program evaluation/  42. ((Program or programme) adj1 evaluation).ti,ab.  43. (Experimental adj1 group$).ti,ab.  44. (Comparative adj1 stud$).ti,ab. or comparative study/  45. (control$ adj1 (trial$ or stud$)).ti,ab.  46. (Before adj1 after adj1 study).ti,ab.  47. exp "randomized controlled trial (topic)"/  48. ((randomized or randomised) adj1 control$ adj1 trial$).ti,ab.  49. controlled study/  50. or/39-49  51. 21 and 38 and 50 | 52. exp animals/ not humans/  53. 51 not 52  54. (comment$ or conference$ or letter or editorial or review).pt.  55. 53 not 54  56. (high adj1 income adj1 country).ti,ab.  57. exp developed country/ or (developed adj1 countr$).ti,ab.  58. 56 or 57  59. 55 not 58  60. (systematic adj1 review$).ti,ab.  61. (meta adj1 analysis).ti,ab.  62. (literature adj1 review$).ti,ab.  63. or/60-62  64. 59 not 63  65. HIV.ti,ab. or exp Human immunodeficiency virus/  66. pregnanc$.ti,ab. or exp pregnancy/  67. exp hypertension/ or hypertension.ti,ab.  68. stroke.ti,ab. or exp cerebrovascular accident/  69. exp cardiovascular disease/  70. malaria.ti,ab. or exp malaria/  71. or/65-70  72. 64 not 71  73. exp rat/ or rat$.ti,ab.  74. mouse.ti,ab. or exp mouse/  75. 73 or 74  76. 72 not 75 |
