# Supplementary material for: Type and extent of trans-disciplinary co-operation to improve food security, health and household environment in low and middle income countries: systematic review
Source: BMC Public Health. 2016 Oct 18;16:1093. doi: 10.1186/s12889-016-3731-4 (PMC5070145; doi:10.1186/s12889-016-3731-4)
Supplement: Additional file 2: — Data Extraction Sheet. (DOCX 21 kb) [file 12889_2016_3731_MOESM2_ESM.docx]

**Additional file 2: Data Extraction Sheet**

| Refwork ID: | | Review Author initials : | |
| --- | --- | --- | --- |
| Title: | | | |
| Authors: | | | |
| Journal: | | | |
| Year of Publication: | | | |
| **Methods** | | | |
| Study Design: | | | |
| Sampling: | | | |
| Year of Study: | | | |
| **Participants** | | | |
| Country: | Income levels: Low Medium | | Ethnicity: |
| Sample size(N): | | | |
| Age: | | Sex: | |
| Sample selection method | | | |
| Inclusion criteria | | | |
| Exclusion criteria | | | |
| **Intervention** | | | |
| Setting: | | | |
| Duration of Intervention: | | | |
| Follow-up: | | | |
| Intervention Details: | | | |
| Intervention I | | | |
| Intervention II | | | |
| Control: | | | |
| **Outcomes** | | | |
| Primary Outcomes: | | | |
| Secondary Outcomes: | | | |
| **Measurement tools:** | | | |
| **Notes:** | | | |

**Results:**

Sample size: Intervention I ________________________________________________________

Intervention II ________________________________________________________

Controlled ________________________________________________________

Age ________________________________________________________

Percentage of Male and female ___________________________________________

Notes ________________________________________________________

| **Outcomes measured** | |  | | | | | | |  |
| --- | --- | --- | --- | --- | --- | --- | --- | --- | --- |
| **Main Findings** : Changes in Specific Outcomes | | | | | | | | | |
| Outcome | Intervention I | |  | Intervention II |  | Controlled |  | *p* | |
|  |  | |  |  |  |  |  |  | |
| 1. Food Productivity |  | |  |  |  |  |  |  | |
|  |  | |  |  |  |  |  |  | |
| 2. Household air quality |  | |  |  |  |  |  |  | |
|  |  | |  |  |  |  |  |  | |
| 3. Dietary diversity |  | |  |  |  |  |  |  | |
|  |  | |  |  |  |  |  |  | |
| 4. Respiratory health |  | |  |  |  |  |  |  | |
|  |  | |  |  |  |  |  |  | |
| 5. Nutritional status |  | |  |  |  |  |  |  | |
|  |  | |  |  |  |  |  |  | |
| 6. Quality of Life |  | |  |  |  |  |  |  | |
|  |  | |  |  |  |  |  |  | |
| 7. Food security |  | |  |  |  |  |  |  | |

**Author’s conclusion:** ______________________________________________________________

______________________________________________________________

______________________________________________________________

______________________________________________________________
